# Supplementary material for: A Macro-meso damage coupling rock mass damage model based on improved internal crack analysis
Source: PLoS One. 2025 Jun 30;20(6):e0326786. doi: 10.1371/journal.pone.0326786 (PMC12208472; doi:10.1371/journal.pone.0326786)
Supplement: Attachment — Submitted filename: sg.pdf [file pone.0326786.s001.pdf]

Main Issues and Recommendations

1. The current assumption that crack propagation direction is always parallel to the principal stress is oversimplified. It is recommended to validate the universality of this hypothesis through multiple comparative experiments.
2. The current model is limited to uniaxial compression conditions. It is suggested to supplement validation experiments under multi-axial stress conditions.
3. Some curves in the figures lack clarity in annotations. It is recommended to add local enlarged views or arrow labels for abnormal regions.
4. Adjust the placement of certain figures to follow their corresponding textual descriptions.
5. The conclusions require stricter phrasing to avoid overgeneralized statements.
6. It is recommended to enhance the discussion on practical engineering applicability.
7. Revise grammatical errors throughout the manuscript and unify the reference format.
8. Supplement citations of critical literature to highlight the novelty and improvements of this work.
