# Supplementary material for: A Macro-meso damage coupling rock mass damage model based on improved internal crack analysis
Source: PLoS One. 2025 Jun 30;20(6):e0326786. doi: 10.1371/journal.pone.0326786 (PMC12208472; doi:10.1371/journal.pone.0326786)
Supplement: Attachment — Submitted filename: Review comments1.pdf [file pone.0326786.s003.pdf]

Review comments:

1、The abstract can be further simplified, highlighting the core innovations and main conclusions of the research;

2、Literature review: it is suggested that more international frontier research should be supplemented and the difference between this paper and existing work should be clarified;

3、It is suggested to explain the actual value of the model with a specific engineering case;

4、It is necessary to clarify the existing research gaps and highlight the innovation of this paper;、

5、It is necessary to further standardize academic expression and correct the problems of text errors, misuse of symbols and inconsistent formats.

This paper introduces an improved macro-meso damage coupling model, demonstrating innovation in theoretical derivation and experimental validation. By refining the literature review, experimental details, and depth of discussion, the manuscript’s academic rigor and application potential will be significantly enhanced。

Recommendation: Accept after minor revisions.
