# Supplementary material for: A Macro-meso damage coupling rock mass damage model based on improved internal crack analysis
Source: PLoS One. 2025 Jun 30;20(6):e0326786. doi: 10.1371/journal.pone.0326786 (PMC12208472; doi:10.1371/journal.pone.0326786)
Supplement: Attachment — Submitted filename: Response to Reviewers-R2.docx [file pone.0326786.s005.docx]

AUTHORS’ RESPONSE TO REVIEWERS’ COMMENTS

Journal:	*PLOS ONE*

Title of the paper: A Macro-meso Damage Coupling Rock Mass Damage Model Based on Improved Internal Crack Analysis

Authors: Haian Liang，Miao He，Hongliang Zhao，Yiru Zhang，Qingrui Lu，Shuren Hao，Zhiying Gong，Hao Guan

Manuscript ID: PONE-D-25-16468

Thank you for your letter and for the reviewers’ comments concerning our manuscript. Those comments are all valuable and very helpful for revising and improving our paper, as well as the important guiding significance to our researches. We have studied comments carefully and have made correction which we hope meet with approval.

*Comment:*

**Point 1:** The abstract can be further simplified, highlighting the core innovations and main conclusions of the research.

**Response:** Many thanks for your advice.

A concise and complete abstract is crucial for an article, as it can help readers quickly identify the main points of the article. Therefore, we have reorganized and rewritten the abstract, highlighting the core part of the research and the research results. (Page 1 lines 8-21 of the revised manuscript).

**Point 2:** Literature review: it is suggested that more international frontier research should be supplemented and the difference between this paper and existing work should be clarified.

**Response:** Many thanks for your advice.

We fully agree with your suggestion to increase international cutting-edge research by citing relevant articles in the article Added references [21,23,24,25,26] in the revised manuscript. and clarifying the differences between this article and existing research. (Page 4 lines 58-66 of the revised manuscript).

**Point 3:** It is suggested to explain the actual value of the model with a specific engineering Case.

**Response:** Many thanks for your advice.

If the established theory is combined with practical engineering, it will have a very positive effect on further enhancing the value of research. However, the study of internal elliptical joints is still mainly focused on the combination of indoor experiments, theoretical experiments, and numerical simulations [1,2,3,4,5], and there is not a lot of relevant engineering practice data. But, in the discussion section of this study, the value and limitations of using constitutive models in practical engineering were discussed. (Page 23-24 lines 321-346 of the revised manuscript). In future research, relevant engineering data will be collected and further validated to improve the practical applicability of the constitutive model, and attempts will be made to address the issues raised in the discussion section.

[1] Yang Y S, Cheng S P, Zhang Z R, et al. Study on the stress field concentration at the tip of elliptical cracks[J]. Reviews on advanced materials science, 2022, 61(1): 611-621.

[2] Niu Z, Wang X, Zhang L, et al. Investigation into the Failure Characteristics and Mechanism of Rock with Single Elliptical Defects under Ultrasonic Vibrations. Fractal and Fractional, 2024, 8(5): 261.

[3] Han Z, Li D, Li X. Experimental study on the dynamic behavior of sandstone with coplanar elliptical flaws from macro, meso, and micro viewpoints. Theoretical and Applied Fracture Mechanics, 2022, 120: 103400.

[4] Liu X R, Yang S Q, Huang Y H, et al. Experimental study on the strength and fracture mechanism of sandstone containing elliptical holes and fissures under uniaxial compression. Engineering Fracture Mechanics, 2019, 205: 205-217.

[5] Aliabadian Z, Sharafisafa M, Tahmasebinia F, et al. Experimental and numerical investigations on crack development in 3D printed rock-like specimens with pre-existing flaws. Engineering Fracture Mechanics, 2021, 241: 107396.

**Point 4:** It is necessary to clarify the existing research gaps and highlight the innovation of this paper.

**Response:** Many thanks for your advice.

We fully agree with your suggestion to clarify existing research gaps and highlight the innovation of this article. Therefore, we have added relevant content in the introduction section of the article to explain the shortcomings of existing research and the prominent characteristics of this study. (Page 3-4 lines 41-66 of the revised manuscript).

**Point 5:** It is necessary to further standardize academic expression and correct the problems of text errors, misuse of symbols and inconsistent formats.

**Response:** Many thanks for your advice.

Standardized grammar is the foundation of an article, so we have optimized the grammar expression and symbol usage throughout the text, unifying the format of the entire text.
